# Heterospecific Dominance Hierarchy in Roosting Site Selection at a Shared Nest Resource

**DOI:** 10.1002/ece3.73081

**Published:** 2026-02-11

**Authors:** Anthony M. Lowney, Robert L. Thomson

**Affiliations:** ^1^ Animal and Agriculture Research Centre Hartpury University Gloucester UK; ^2^ FitzPatrick Institute of African Ornithology University of Cape Town Cape Town South Africa

**Keywords:** ecosystem engineers, Kalahari, resource limitation, social hierarchy, thermal refugia

## Abstract

The resources needed by different species are fundamental for allowing multiple species to coexist. However, when species share resources, competition is expected to occur with associated costs. Sociable weavers ( *Philetairus socius* ) build large communal nests that provide, among other resources, nesting chambers that provide shelter, protection, thermal buffering and insulation for roosting birds of other species. We consider the interactions of heterospecifics roosting in colonies to determine if species select chambers due to their insulation properties, if there is a dominance hierarchy in acquiring chambers, and/or if novel behaviours to access chambers are employed. Our study demonstrates that different species use different aspects of the nest resource, preferring roosting chambers depending on the location of the chamber within the colony. To access this resource, we show that aggressive interactions occur between the species, resulting in a dominance hierarchy with size being positively related to the dominance of a species. Furthermore, our data show temporal separation in timing of arrival at the chambers, with smaller species tending to arrive later and occupy vacant chambers, thus avoiding aggressive interactions with more dominant heterospecifics. Therefore, adapting to competition, multiple species use novel behaviours and interactions, allowing them to coexist at this same engineered resource.

## Graphical abstract

Our study demonstrates that different species use different aspects of the nest resource, preferring roosting chambers depending on the location of the chamber within the colony. To access this resource, we show that interactions occur between the species, resulting in a dominance hierarchy with size being positively related to the dominance of a species. Furthermore, our data show temporal separation in timing of arrival at the chambers, with smaller species tending to arrive later and occupy vacant chambers, thus avoiding aggressive interactions with more dominant heterospecifics. Therefore, adapting to competition, multiple species use novel behaviours and interactions, allowing them to coexist at this same engineered resource.

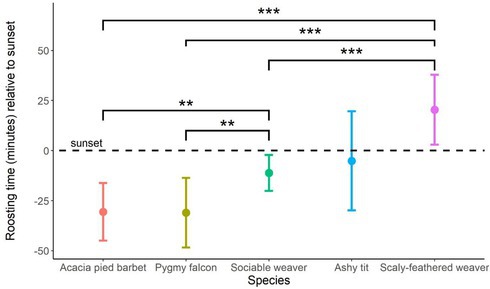

## 1 Introduction

Resources contribute to individual survival and reproductive output (Tilman 1982). These include food, habitat (Begon et al. 1986), shelter (Cole et al. 2005), sexual partners (Danchin et al. 2008) and even social information between individuals (Seppänen et al. 2007). When species share the same resources and these are limited, competition is expected to occur with associated costs (Blaustein 1980; Knight et al. 2004; Zeng and Lu 2009). Animals can gain access to resources through aggressive interactions (Clutton‐Brock et al. 1979; Bergman and Moore 2003), which could cause energetic costs and risk injury or death (Clutton‐Brock et al. 1979; Neat et al. 1998; DeCarvalho et al. 2004).

The ability to compete for resources is often linked to animal size, with larger individuals usually winning aggressive interactions (Persson 1985; Francis et al. 2018). Dominance hierarchies may form which can reduce aggressive interactions (and associated costs) between individuals or species (Smith 1974; Drews 1993). This research has largely focussed within an intraspecific context (Wittig and Boesch 2003; Archie et al. 2006); however, mechanisms determining rank of heterospecifics in dominance hierarchies will have important ecological consequences such as community structure, because dominant species tend to have greater access to certain resources (Zeng and Lu 2009). Therefore, hierarchical position may have survival or reproductive consequences (Francis et al. 2018), and understanding these interactions in different systems will provide insight in community processes.

In contrast to competitive interactions, some species facilitate the availability of resources in systems by ameliorating conditions or creating habitat (Coggan et al. 2018). In some cases, species can dramatically alter the availability of resources leading to changes in the local communities (Flecker 1996; Crooks and Khim 1999; Bancroft et al. 2008; Natusch et al. 2016), impacting community structure and ultimately increasing community species richness (Bertness and Callaway 1994; Wright et al. 2002; Castilla et al. 2004; Badano and Cavieres 2006). Species that have disproportionately high effects on the communities and the ecology of an ecosystem are often termed ecosystem engineers (Jones et al. 1994; Badano and Cavieres 2006). While the net effect of this facilitation may be positive, species in these communities, including the ecosystem engineer, may experience increased competition and predation. By adapting to competition, multiple species can use novel behaviours and interactions, allowing them to coexist at the same engineered resource (Schemske et al. 2009).

Sociable weavers ( *Philetairus socius* ) are endemic to the semi‐arid and arid Kalahari of southern Africa and build large colonial nest structures (Maclean 1973a; Mendelsohn and Anderson 1997). Sociable weavers have been shown to enhance local diversity and as such classed as ecosystem engineers (Lowney and Thomson 2021, 2022; Buckley and Maritz 2025). They have been shown to enhance soil properties and influence local vegetation (Prayag et al. 2020; Aikins et al. 2023). Greater abundance and diversity of multiple taxa have been demonstrated at trees with a colony than those without, including invertebrates (Lowney and Thomson 2022), reptiles (Rymer et al. 2014; Lowney and Thomson 2022; Buckley and Maritz 2025), birds, and large and small mammals (Lowney and Thomson 2022, 2021). These colonies also remain in the landscape for decades and as such become landmarks in the local environment and are used frequently for scent marking by large mammals including cheetahs ( *Acinonyx jubatus* ; Lowney and Charlton 2017). The importance of these resources has been shown to increase in harsher environments (Lowney and Thomson 2022), with unique behaviours between interacting species at colonies being observed (Lowney, Bolopo, et al. 2020; Lowney, Flower, and Thomson 2020; Lowney and Thomson 2024). Therefore, understanding the competition and interactions between species using sociable weaver colonies will enable a greater understanding of the potential influence in the ecological role of weavers and their colonies.

Each colony contains multiple nesting chambers and can vary in size, ranging from a few to over 250 chambers, each with its own entrance tunnel that opens up to the bottom of the colony (Maclean 1973a). The Kalahari environment presents extreme temperatures that can surpass the upper and lower thresholds of many species in animal communities persisting in these harsh landscapes. Winter temperatures can force animals to conserve energy by initiating hypothermia (McKechnie and Lovegrove 2002) while temperatures in summer have the potential to cause lethal hyperthermia (Cunningham et al. 2013; Andreasson et al. 2019). Sociable weavers use and maintain the colonies year‐round, both during breeding and non‐breeding periods, when they use the chambers for roosting at night (Lowney, Bolopo, et al. 2020; Lowney, Flower, and Thomson 2020). Colonies provide clear thermal refugia from external temperatures as they are cooler in summer and warmer in winter (Van Dijk et al. 2013; Leighton and Echeverri 2014; Lowney, Bolopo, et al. 2020). Chambers towards the centre of the colony provide greater thermal buffering than those on the edge (Lowney, Bolopo, et al. 2020), and this affects the social dynamics of sociable weavers with dominant individuals occupying the better‐insulated central chambers (Van Dijk et al. 2013). Furthermore, when weavers are not breeding and external temperatures are low, multiple individuals will roost in a single chamber, this further increases the nest temperature reducing the effects of cold ambient temperatures (Paquet et al. 2016; Lund et al. 2020).

The insulating properties of chambers within sociable weaver colonies also provide thermally buffered chambers for small bird species (Maclean 1973b; Lowney and Thomson 2021, 2022). Several bird species regularly use weaver chambers for roosting (Maclean 1973b; Lowney and Thomson 2021), and some of these also use them for breeding (Maclean 1970; Ndithia et al. 2007; Olubodun et al. 2023). Five species commonly use sociable weaver chambers in the southern Kalahari (Lowney and Thomson 2021); African pygmy falcon ( *Polihierax semitorquatus* ), Acacia pied barbet ( *Tricholaema leucomelas* ), ashy tit (*Melaniparus cinerascens*), red‐headed finch ( *Amadina erythrocephala* ) and scaly‐feathered weaver ( *Sporopipes squamifrons* ). These associate species coexist with sociable weavers in their colonies, and can cause costs for their sociable weaver hosts, for example, African pygmy falcons are known to predate sociable weaver nestling, fledglings and even adults (Covas et al. 2004; Lowney and Thomson 2024). Anecdotal observations also show conflicts around the colony between the hosts and Acacia pied barbet (personal observations). Temperature‐buffered chambers in the weaver colonies, provide an interesting case of resource competition where interspecific dominance hierarchies within a community may form, and where positions in the hierarchy may have fitness consequences and ultimately effect species local adaptation and life history evolution. For example, competition for chamber towards the centre of the colony should be greater than those at the edge, but nothing is known about the interactions and hierarchies within the avian community that uses these colonies.

Here we aimed to understand the interactions between bird species that use sociable weaver colonies as a resource for night‐time roosting. We investigate if heterospecifics prefer certain chambers due to their insulation quality and if an interspecific dominance hierarchy allows certain species to acquire the chambers they choose. Previous studies that have investigated dominance hierarchies in bird communities have largely looked at competition for food resources (Miller et al. 2017; Francis et al. 2018), but few to none have looked at competition for roosting resources. We firstly describe characteristics of roost use, such as the roosting chamber location used by heterospecific species and behavioural variables related to timing of arrival for roosting. We then experimentally altered roost chamber availability and recorded subsequent choices, displacements and aggressive interactions between heterospecifics when competing for chambers. We hypothesised that there would be competition for roost sites, with a dominance hierarchy among the species using the chambers and that the more dominant species would access the more central chambers; however, this may not be true for pygmy falcons as these birds are larger and tend not to roost in central chambers, due to the entrances being smaller and more compact (Lowney, Bolopo, et al. 2020). We also hypothesised that to avoid aggressive interactions, we expected temporal separation of roosting times, with least dominant individuals avoiding conflict costs by arriving at roosts later, and acquiring vacant chambers, least preferred chambers.

## 2 Materials and Methods

### 2.1 Study Site and Species

This study was undertaken at Tswalu Kalahari, an approximately 1300 km^2^ reserve in the Northern Cape, South Africa (27°13′30″ S and 22°28′40″ E). Our study area within Tswalu Kalahari spans approximately 130 km^2^ and contains over 250 active sociable weaver colonies built mainly on camelthorn ( *Vachellia erioloba* ) and shepherd's tree (*Boscia albitrunca*) (Aikins et al. 2023; Olubodun et al. 2023). The local climate is hot and arid with mean annual temperatures of 16.8°C–18.2°C that can drop below freezing in winter and can exceed 40°C during summer (Lowney, Bolopo, et al. 2020).

### 2.2 Survey Methods

To understand species use of chambers within sociable weaver colonies and the hierarchy between species, we designed a 5‐day trial at colonies that contained at least one heterospecific roosting in a chamber overnight. Between September and December 2017, we monitored 56 sociable weaver colonies for this study. No breeding occurred in any of these colonies during this study which was checked by inspecting chambers with an extendable mirror every few days during the trials. We worked with five different colonies at a time during trials that lasted 5 days. To select the colonies, we surveyed colonies in a specific area by visiting colonies at night and used a head torch to scan colony chambers for heterospecifics (species that were not a sociable weaver). We carried this out 30 min after local sunset as this prevented the flushing of individuals from a given colony (Lowney and Thomson 2021, 2022). Any birds occupying the chambers were generally visible and could be identified to species. When we found five colonies that contained at least one chamber occupied by a heterospecific, we used these for the current trial. After these 5 days, we moved to five new colonies, slowly moving around the study area until most colonies had been surveyed. A total of 49 colonies were used in trials.

First, we gathered data on the number of heterospecifics per chamber. The number of heterospecifics per chamber was documented during survey on night one. Here, due to the large number of colony chambers (up to 235 in this study) and the large number of sociable weavers (> 400 in some colonies), we focused mainly on the heterospecifics as it was impractical to count all the individuals in all the chambers. Second, the time‐of‐day sociable weavers and heterospecifics roosted (minutes relative to sunset) was determined using video cameras set up at the colonies on day three of the trial (night before the chamber blocked, see below). Roosting time was taken as the time when individuals entered the chambers and did not leave for the night. Sociable weavers in the same colony may roost at different times; therefore, we defined roosting time as the time when the first individuals entered their chambers and did not leave. Roosting times of sociable weavers were taken from the same recordings as the roosting times for the other heterospecifics and were therefore paired in our statistical analyses.

Some colonies had more than one chamber occupied by heterospecifics. To remove any confounding factors that multiple trials at the same colony may add, we only selected one heterospecific‐occupied chamber per colony. When two or more chambers were occupied by the same heterospecific species, we would randomly choose which individual to monitor. For colonies hosting more than one heterospecific species, we selected the species that were less frequently observed to use colonies (as identified in Lowney and Thomson 2021), which was done to increase our sample size for these species. Once an individual was observed, the number of individuals in the chamber and the chamber location were recorded. Chamber location was designated in three categories: those near the edge, those near the centre, and those mid‐way between the edge and the centre chambers (Lowney, Bolopo, et al. 2020).

We returned on the second and third nights to determine if individuals were using the same chamber. Pygmy falcons in this population are individually marked, with a unique colour‐ring combination as part of an ongoing study (Bolopo et al. 2019; Olubodun et al. 2023); therefore, we were able to determine if the same individuals returned to the same chambers. However, all other species were unmarked, but we assume heterospecific individuals returning to the same chamber were the same individuals. This assumption should be overall robust due to the territoriality of many of these species (Lowney et al. 2017; Kopij 2023) and the low densities of most species in the arid desert environment. In addition, with colonies containing potentially hundreds of distinct chambers (up to 235 in this study), the probability of different heterospecifics using the same chamber would be low. Therefore, if a heterospecific individual(s) was observed using the same chamber for multiple nights, then it was assumed to be the same individual(s). If an individual of a species was not seen using the same chamber as the previous night, we would note the species of the new occupant, and the trial at that colony was ended.

### 2.3 Experimental Trials

If the same species (assumed individual) was recorded using the same chamber for all three initial days, then on the fourth day, we created an experimental treatment by blocking this chamber using chicken wire. On the fourth night, we would survey the colony as described above and record where this species moved for that night, for example, whether it roosted in a previously empty chamber, replaced another species, joined a conspecific, or spent the night away from the colony.

To confirm movements of focal individuals (those that had their chamber blocked) and to understand the competitive behaviours at colonies, we used video cameras (Canon Legria HF R806) placed from day two onwards and were set facing up towards the chambers but zoomed out enough so that all chambers of the weaver colony were in view. For larger colonies, multiple video cameras were used. Video cameras showed the individual trying to enter its original roosting chamber before roosting elsewhere and recorded any aggressive interactions (birds chasing other birds away) with other individuals/species. If a heterospecific was observed roosting in a different chamber on the night that the chamber was blocked, we would use the recordings from the previous nights to determine which species, if any, had previously occupied the new chamber. On the final day, we would unblock the chamber and monitor to see if the individual returned to the original chamber or remained where it roosted on night four.

Ethical note: Aggressive interactions between heterospecifics coming to roost at sociable weaver colonies are commonly observed. In addition, despite blocking the roosting chambers of focal individuals, the high density of sociable weaver colonies across our study site, combined with the high number of chambers in each colony (mean 40 chambers, range 14–235), means that there are many other chambers that were still available.

To determine if a dominance hierarchy exists between species, we used the (1) information of species replacing others, and (2) any aggressive interactions observed from video recordings on the evening the chambers were blocked. The ‘winner’ of a dominance interaction was defined as the species that moved into a new chamber that had previously been occupied by another species. A ‘loser’ was defined as the species that was replaced. Videos from the evening when chambers were blocked were also used to determine if the focal species tried to enter another chamber but was chased away. If an individual was unsuccessful and chased away, these were also defined as a ‘loser’, and the species that defended the chamber, as the ‘winner’.

### 2.4 Statistical Analysis

We analysed all data using R statistical package 4.1.3. (R Core Team 2017), using the glmmTMB package (Brooks et al. 2019). We explored statistical differences using pairwise post hoc tests in the emmeans package (Lenth 2018). Firstly, we compared the number of heterospecifics (non‐sociable weavers) roosting in a chamber using a Poisson‐distributed GLM, due to the dispersion of the count data (dividing the residuals of freedom by the deviance). The number of individuals was used as the response variable, and species was the only explanatory variable. All model details are listed in Table A1.

To compare roosting times (start of roosting) among species, we used a LMM with roosting time as the response variable and species as the explanatory variable. Roosting time was calculated in minutes in relation to the sunset. Species was used as the only explanatory variable. The roosting times of focal heterospecifics and sociable weavers were taken from the same colony; therefore, each colony was given a unique ID that was used as a random factor.

To infer the dominance hierarchy and estimate associated uncertainty in species rankings, we used the randomised Elo‐rating method (Sánchez‐Tójar et al. 2018), using the ‘aniDom’ package (Farine and Sanchez‐Tojar 2018). To determine the robustness of the between‐species hierarchy estimation, we calculated the repeatability score for species ranks based on the randomised Elo ratings (Sánchez‐Tójar et al. 2018). To determine if body mass influenced the dominance ranking, we used a Pearson's correlation to compare a species' average weight against their dominance ranking. Average body mass of the bird species included in the trials was taken from Chittenden (2007). We used descriptive statistics (frequencies) to compare the original chamber selection by each species. This is for periods when the chambers remained accessible and when they were blocked.

## 3 Results

In total, 49 trials were carried out, with 45 fully completed for the full 5‐day protocol (Table 1). The four abandoned trials were due to three occasions when the focal chamber was occupied by a different species on nights two or three, and on one occasion, the heterospecific individual did not return to the colony to roost.

**TABLE 1:** The number of attempted and completed full 5‐day trial for each species.

Species	Trials	Complete	Displaced	Did not return
Acacia pied barbet	20	18	1	1
Ashy tit	8	7	1	0
Pygmy falcon	10	10	0	0
Scaly‐feathered weaver	11	10	1	0

### 3.1 Number of Individuals in a Focal Chamber

A total of 144 heterospecific individuals were observed using focal chambers (mean 3.2 ± SD 3.99). The number of individuals roosting in a chamber varied by species, with Acacia pied barbets (*n* = 18) and ashy tits (*n* = 7) always roosting on their own, while on average, 1.7 pygmy falcons (range 1–2; *n* = 10) and 10.2 scaly‐feathered weavers (range 7–14; *n* = 10) were observed roosting in a chamber together.

**FIGURE 1:**
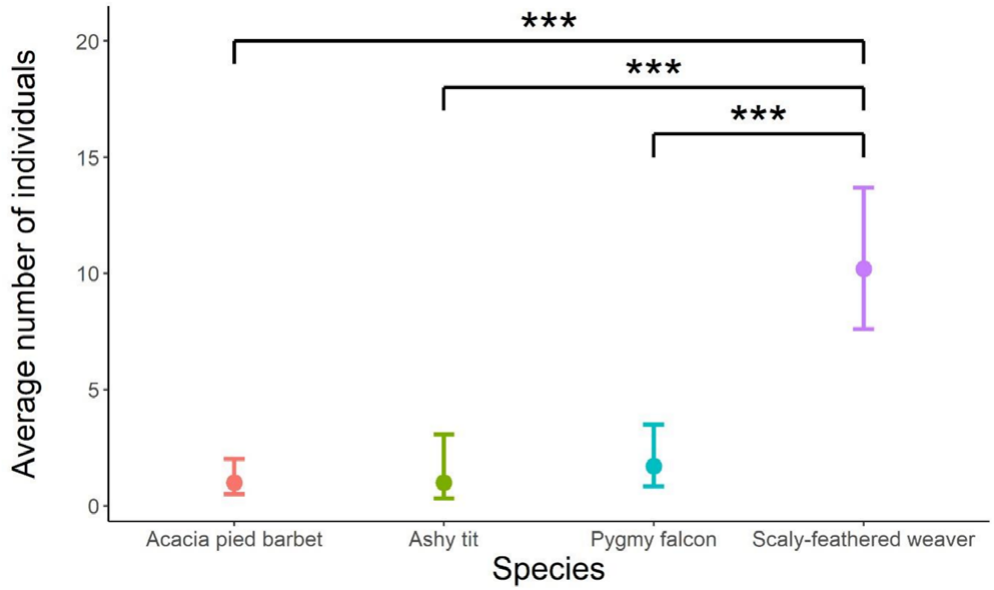
The average number of individuals roosting in a sociable weaver chamber on the first night of the trials. Post hoc comparisons revealed significant between‐species differences in the numbers of individuals roosting in a chamber (***p < 0.001).

Species explained significant variation in the number of individuals in a chamber (*χ* ^2^ = 135, *p* = < 0.001). A post hoc comparison revealed that the number of scaly‐feathered weaver individuals roosting together was significantly greater than Acacia pied barbet (*t* = −9.084, *p* = < 0.001), ashy tit (*t* = −5.944, *p* = < 0.001), and pygmy falcon (*t* = −6.84, *p* = < 0.001; Table A2, Figure 1) in focal chambers. No differences were observed between the other species.

### 3.2 Roosing Times

**FIGURE 2:**
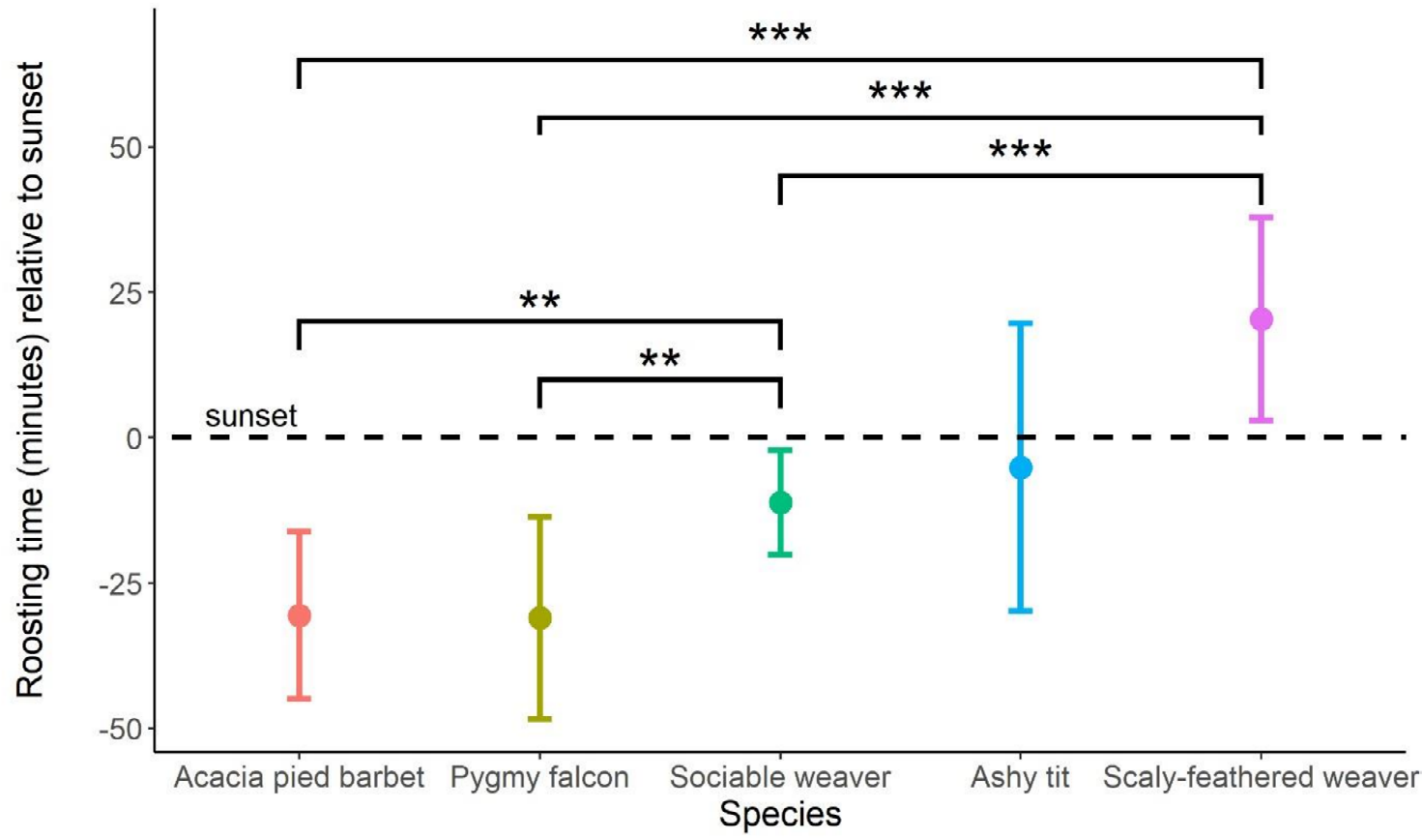
Mean roosting times in comparison to local sunset. Negative numbers indicate a species roost before sunset. Positive numbers indicate that a species roosts after sunset. Post hoc comparisons revealed significant between‐species differences in the numbers of individuals roosting in a chamber (**p < 0.01; ***p < 0.001).

On average, sociable weavers and heterospecifics roosted at 13.09 (±2.72 SE) minutes before sunset. Roosting time differed among species (*χ* ^2^ = 65.311, *p* = < 0.001). The earliest roosting time was recorded as 94 min before local sunset and the latest roosting time being 48 min after sunset. African pygmy falcons and Acacia pied barbets showed the earliest roosting times, on average 30.99 min (±5.78 SE) and 30.56 min (±4.76 SE) before sunset, respectively. Sociable weavers and ashy tits roosted on average 11.16 (±2.97 SE) and 5.11 (±8.17 SE) before sunset, respectively, while the scaly‐feathered weaver roosted at 20.44 (±5.78 SE) minutes after sunset. Acacia pied barbets (*t* = −4.01, *p* = 0.001) and pygmy falcons (−3.42, *p* = 0.009) roosted significantly earlier than sociable weavers (Figure 2) and significantly earlier than the scaly‐feathered weaver (*t* = −7.25, *p* = < 0.001; *t* = −6.46, *p* = < 0.001, respectively; Figure 2; Table A3). Sociable weavers roosted 31 min before the scaly‐feathered weaver (*t* = −5.40, *p* = < 0.001, Figure 2).

### 3.3 Dominance

During the 49 trials, three of the species were replaced by a heterospecific on at least one occasion; these were the Acacia pied barbet, ashy tit, and scaly‐feathered weaver. Pygmy falcons were never displaced from their roosting chamber by another species. When their roosting chambers were experimentally blocked, Acacia pied barbets displaced a heterospecific on nine occasions but failed to do so on 10 occasions (Table 2). On six of these 10 occasions, sociable weavers were observed to successfully defend their chambers against the barbets. In contrast, when roosting chambers of ashy tits and scaly‐feathered weavers were experimentally blocked, these species failed on all occasions to roost in a new chamber that had been occupied by any other species the day before. On two occasions, ashy tits tried to enter chambers that had hosted sociable weavers on the previous night, but they were chased away on both occasions. Scaly‐feathered weavers either roosted with conspecifics or elsewhere. Pygmy falcons displaced sociable weavers on four occasions, joined conspecifics on three occasions, and used an empty chamber on the other three occasions (Table 2).

**TABLE 2:** Summary of interspecific aggression, showing the number of trials, which of the focal species were displaced by others before the chamber was blocked, and when the chamber was blocked who joined a conspecific, who replaced a heterospecific, who moved to an empty chamber, or roosted elsewhere.

Species	Number of trials	Displaced by heterospecific	Joined conspecific	Replaced heterospecific	Moved to empty chamber	Another colony/outside
Acacia pied barbet	20	1	2	9	4	4
Ashy tit	8	1	0	0	6	1
Pygmy falcon	10	0	3	4	3	0
Scaly‐feathered weaver	11	1	4	0	0	6

In total, we observed 25 occasions when either an individual replaced a heterospecific individual or was observed participating in aggressive interactions with heterospecifics attempting to gain access to a particular chamber. Our analysis found that pygmy falcons were estimated to be the most dominant species, followed by Acacia pied barbet and sociable weaver, while ashy tits and scaly‐feathered weaver were the least dominant (Table 3). Species *Elo* scores were highly repeatable (*r* = 0.889), demonstrating a high degree of confidence in the hierarchy estimation and that the rank largely predicts the probability of winning an interaction. As predicted, dominance was found to be significantly associated with body mass (Pearson's correlation −0.918, *p* = 0.02).

**TABLE 3:** Summary of the species, body mass and dominance rank of all birds observed participating in dominance interaction at sociable weaver colonies, in order of decreasing dominance.

Species	Body mass (g)	Dominance rank	95% confidence range
Pygmy falcon	60	1.00	1–1
Acacia pied barbet	31	2.55	2–4
Sociable weaver	27	2.63	2–4
Scaly‐feathered weaver	11	4.18	3–5
Ashy tit	20	4.64	3–5

*Note:* The 95% confidence intervals of the dominance rank associated with each estimate of dominance rank are included as an indicator of uncertainty, estimating using randomised Elo‐ratings. Species body masses were obtained from Chittenden (2007).

### 3.4 Chamber Selection

Overall, Acacia pied barbets were observed using central chambers more frequently (12 of 18 occasions, Table 4). However, when these chambers were blocked, they were found to use intermediate chambers most frequently (8 of 18 occasions, Table 4). Scaly‐feathered weavers were found in chambers on the outside edge of the colony in all observations, and on five occasions when the focal chamber was blocked, they failed to access a chamber at the focal colony (Table 4). Pygmy falcons were observed using outside edge and intermediate chambers more frequently than chambers in the centre of colony (Table 4) and maintained the same frequency of chamber choice after experimental blockage. Ashy tits were seen using the intermediate chambers more frequently than the centre and the edge chambers (Table 4). On Day 5 of the trials, when the blockage was removed, scaly‐feathered weaver always returned to roost in the original chambers, ashy tits returned six out of the seven trials, Acacia pied barbet 9 of 18 trials and pygmy Falcons six out of the 10 trials.

**TABLE 4:** Summary of chamber location used by each of the focal species before and during the original roosting chamber being blocked.

Species	Chambers all open			Focal chamber blocked			
	Centre	Intermediate	Edge	Centre	Intermediate	Edge	Another colony/outside
Acacia pied barbet	12	4	2	4	8	4	2
Ashy tit	2	4	1	0	4	2	1
Pygmy falcon	1	4	5	1	4	5	0
Scaly‐feathered weaver	0	0	10	0	0	5	5

## 4 Discussion

Our study demonstrates that different species associated with sociable weaver colonies use chambers in different locations within the colony. To access the ‘chamber‐resource’, we also show that aggressive interactions occurred between the species, resulting in a dominance hierarchy which had consequences for the choices of the type of roosting resource (chamber location) that the species most frequently uses. Furthermore, our data show temporal separation in timing of arrival at the chambers, with smaller species tending to arrive later and take what is available. This may be due to species‐specific foraging rhythms and/or to avoid aggressive interactions with more dominant heterospecifics. We found pygmy falcons to be the most dominant species, choosing chambers close to the edge of the colony. Scaly‐feathered weaver arrived last and used outside chambers that were unoccupied. Whereas for the other species there was an increased use/preference for central chambers, especially for Acacia pied barbets, with competition for these chambers intense and this species involved in the greatest number of aggressive interactions trying to access these chambers.

### 4.1 Dominance

Our study demonstrates that a dominance hierarchy exists between the species that roost in sociable weaver chambers. As with other studies, this hierarchy is positively associated with body mass (Francis et al. 2018), with the larger species being the more dominant. Although the species were observed to use different types of chambers, the smaller species likely do not have access to preferred chambers due to the aggression from more dominant species. Our results suggest that scaly‐feathered weavers may be more dominant than ashy tits, a species nearly twice their size. However, this is unlikely and probably driven by the reduced number of interactions observed between these two species. Scaly‐feathered weavers only use large entrance edge chambers which are seldom used by other species. Neither species was seen to displace any other heterospecific. The accessibility of the ‘chamber‐resource’ was therefore determined by which species were associated with a particular colony. Species lower in dominance hierarchy, ashy tit and scaly‐feathered weaver, would be unable to utilise the resource if all chambers were occupied by other species. Probably driven by their low position in the dominance hierarchy resulting in poor access to weaver colonies, ashy tits and scaly‐feathered weavers were also the least frequently observed using weaver colonies, see also Lowney and Thomson (2021).

Pygmy falcons were the most dominant species and were the only species not to be displaced. In addition, no aggressive interactions were observed involving pygmy falcons. When falcons approached colonies all other species dispersed, only roosting once the falcons had roosted. Falcons are substantially larger than the other species, being twice as heavy as the second and third largest species within this study (Table 4; Chittenden 2007). Pygmy falcons are predators and, although their main prey consists of small reptiles and insects (Maclean 1970), they are known to prey on mammals and small birds including sociable weavers (Maclean 1970; Spiby 2014; Lowney and Thomson 2024), and scaly‐feathered weavers (personal observations). Therefore, Acacia pied barbets, ashy tits, sociable weavers, and scaly‐feathered weavers are unlikely to interact aggressively towards falcons or replace them in falcon‐occupied chambers.

Acacia pied barbets and sociable weavers had similar dominance ranks (Table 3), and interactions between these two species were the most frequently observed. Both species won aggressive interactions for access to chambers a similar number of times. Barbets were slightly larger than sociable weavers and, as a result, would be expected to ‘win’ aggressive interactions (Francis et al. 2018). However, numbers of sociable weavers are much higher at colonies compared to barbets, and barbets often need to deal with groups of sociable weavers chasing barbets away from chambers in the colony (personal observations). Therefore, multiple individuals defending chambers may allow sociable weavers to win aggressive interactions against a slightly larger competitor.

### 4.2 Timing and Location

The larger species (barbets and falcons) started roosting earlier and the smaller species later. The earliest a barbet was observed roosting was more than an hour and a half before sunset; this was over an hour before all other species. Early arrival allowed barbets access to colony chambers with less probability of aggressive interactions with sociable weavers, and likely greater access to central chambers which were most used/preferred by this species. Central chambers offer greater insulation (Leighton and Echeverri 2014; Lowney, Bolopo, et al. 2020) and are likely safer from predators. Indeed, these central chambers are also preferred by dominant sociable weavers (Van Dijk et al. 2013). In addition to early arrival, barbet success at acquiring central chambers appeared to be an ability to remember the location of their target chamber and fly directly to it when weavers were present. However, when a chamber was blocked, the barbet would often get to the entrance before being chased away. On these occasions, it would often be chased away before it could investigate other chambers, meaning that it would not always be able to roost in a chamber in its preferred location. Once in the chamber, weavers could not evict barbets (personal observations).

Pygmy falcons roosted before most other species and preferred chambers at the edge of the colony. Previous research revealed falcon preference for chambers at the edge of colonies (Lowney, Bolopo, et al. 2020), which appears to be because central chambers have longer entrance tunnels that are more difficult (or impossible) to access (Lowney, Bolopo, et al. 2020). The reasons for early initiation of roosting by falcons are uncertain because we found no evidence of aggressive interactions with weavers. We found that weavers do not roost until the falcons have. When falcons approach colonies, sociable weavers alarm and flee (Lowney, Flower, and Thomson 2020). Indeed, weavers that had already started roosting or were in the process of roosting also flee in response to alarm calls generated due to arriving falcons. Falcons have been observed investigating weaver chambers and eating any chicks that may be inside (Maclean 1970; Lowney and Thomson 2024), and adult weavers would likely avoid the risk of being cornered in a chamber when a falcon arrives at a colony. Further studies should be carried out to determine whether weavers that do not share colonies with falcon's roost earlier and if so whether this has an impact on the birds sleep and overall condition.

Roosting times may differ due to species‐specific diurnal activities (Capilla‐Lasheras et al. 2025). However, communal roosting species with similar feeding habitats have been shown to roost at similar times (Morrison and Caccamise 1990). Acacia pied barbets, sociable weavers, ashy‐tits, and scaly‐feathered weavers all share some overlap in the foods they consume (Chittenden 2007), and may suggest that if not for the competition for roosting chambers, then these species may roost at similar times, and that avoidance of aggressive interactions may be a reason behind the differences in the times observed. Although we cannot rule out that different diurnal foraging patterns may lead to the differences in roosting times observed in these species. Pygmy Falcons feed mainly on small reptiles. The activity of these prey species may dictate when falcons roost due to the reduced activity during lower temperatures by reptiles (Zeng et al. 2025).

Ashy tits tended to use chambers between the centre and the edge of the colony. While scaly‐feathered weavers were always seen using chambers on the outside edge of the colony. It is likely that both species may prefer the chambers with better insulation, but due to being low on the dominance hierarchy both species roost later and take chambers that are available at after sunset. Ashy tits have been observed roosting in chambers previously occupied by pygmy falcons, those with faecal ‘mats’ around the entrance (Krochuk et al. 2018), something sociable weavers rarely tend to do (personal observations). By using old, marked falcon chambers, ashy tits may be able to access colony chambers that are located between the centre and the edge of the colony, which provide better insulation than chambers at the edge of the colony (Lowney, Bolopo, et al. 2020).

### 4.3 Number of Individuals

Acacia pied barbets and ashy tits were largely observed roosting alone, while pygmy falcons and scaly‐feathered weavers were seen roosting with conspecifics. As both falcons and scaly‐feathered weaver tended to roost in chambers on the edge of a colony, sharing a chamber with conspecifics may reduce the impacts of spending the night in chambers with poorer insulation. We observed no more than two falcons in a chamber; however, during winter, up to three falcons can be seen roosting together (Lund et al. 2020), suggesting that more individuals share chambers during colder ambient temperatures for the thermoregulatory benefits of group roosting on cold winter nights (Paquet et al. 2016). Scaly‐feathered weavers benefit from roosting together, by reducing energy expenditure while maintaining their body temperature at night. Using weaver nest chambers reduces energy expenditure further (Lubbe et al. 2018). Both Pygmy Falcons and scaly‐feathered weavers are social species (Lubbe et al. 2018; Bolopo et al. 2019), and this may allow them to tolerate being near to other individuals compared to the barbets and ashy tits. Sociable weavers also share their chambers with conspecifics, with up to eight individuals roosting in a single chamber when external temperatures are low, and they are not breeding (Paquet et al. 2016). This further increases the nest temperature reducing the effects of the cold ambient temperatures (Lubbe et al. 2018).

### 4.4 Limitations

Despite our confidence in the interpretation of our findings, there are limitations within this study. Firstly, the sample size of certain species is somewhat small, yet clear differences and signals were apparent from the data, suggesting more trials would just add unnecessary disturbance. It was also impractical to block chambers that sociable weavers were using to roost because, with potentially hundreds of unmarked weavers per colony, gaining choice or consequence data was not possible. Lastly, the lack of individually marked heterospecific individuals (excluding pygmy falcons) means individual decisions are assumed, but this assumption seems robust as individuals of these species are territorial (Lowney et al. 2017; Kopij 2023), while the probability of observing the same species using the same chamber night after night, despite low bird densities and an abundance of chambers in the environment, seems very unlikely.

## 5 Conclusion

Our study agrees with previous research demonstrating the importance of weaver colonies as a resource to other bird species (Maclean 1973b; Lowney and Thomson 2021, 2022). We demonstrate competition between species for access to sociable weaver colony chambers, which leads to a dominance hierarchy and that the ability to compete differs between species. The larger species tend to be the more dominant, gaining greater access to resources, while the less dominant species tend to avoid heterospecific interactions. The dominance position of a species in this community influences accessibility to temperature buffered chambers, and the presence of dominant heterospecifics may ultimately have negative fitness consequences (Francis et al. 2018). When the dominance hierarchy is less clear, aggressive interactions occur. Behavioural adaptations such as timing of initiation of roosting allowed species to gain access to preferred chambers and/or avoid conflict with more aggressive heterospecifics.

By building a physical habitat that provides resources, sociable weavers positively impact local species assemblages and community structure (Lowney and Thomson 2021, 2022), and although the net effect of this facilitation may be positive, species in these communities, including the ecosystem engineer, experience competition to access colony chambers. Therefore, adapting to competition, multiple species use novel behaviours and interactions, allowing them to coexist at this same engineered resource.

## Author Contributions

**Anthony M. Lowney:** conceptualization (equal), data curation (equal), formal analysis (equal), investigation (equal), methodology (equal), project administration (equal), writing – original draft (equal), writing – review and editing (equal). **Robert L. Thomson:** conceptualization (equal), formal analysis (equal), funding acquisition (equal), methodology (equal), project administration (equal), resources (equal), supervision (equal), writing – original draft (equal), writing – review and editing (equal).

## Ethics Statement

Ethics approval from the University of Cape Town, South Africa (2015/V14/RT).

## Conflicts of Interest

The authors declare no conflicts of interest.

## Supporting information


**Video S1:** Sociable weavers chasing away an Acacia pied barbet from a sociable weaver colony.

## Data Availability

Data are deposited in ZivaHub Digital Repository https://figshare.com/s/0f5dfcb9e6b9124b0be8.

## Appendix A

**TABLE A1:** Complete list of model structure used.

Response variable	Model	Distribution	Explanatory variables	Random effects
Number of individuals	GLMM	Poisson	Species	
Minutes compared to sunset	LMM	Gaussian	Species	Colony ID

*Note:* Each response variable is listed and the full list of explanatory variables, random effects, error distribution used. All models were run in the glmmTMB package using the glmmTMB function (Brooks et al. 2019).

**TABLE A2:** Post hoc analyses demonstrate that the differences between the number of individuals in a focal chamber.

Contrasts	Odds ratio	±SE	*t*	*p*
Acacia pied barbet—Ashy tit	1.000	0.445	0.000	1.000
Acacia pied barbet—Pygmy falcon	0.588	0.199	−1.569	0.396
Acacia pied barbet—Scaly‐feathered weaver	0.098	0.025	−9.084	< 0.001
Ashy tit—Pygmy falcon	0.588	0.264	−1.182	0.639
Ashy tit—Scaly‐feathered weaver	0.098	0.038	−5.944	< 0.001
Pygmy falcon—Scaly‐feathered weavers	0.167	0.044	−6.840	< 0.001

*Note:* Scaly‐feathered weavers and all other species explain much of the variation (*p =* < 0.001).

### Roosting Times

**TABLE A3:** Post hoc analyses demonstrate that a variety of species roosting times explain much of the variation.

Contrasts	Estimate	±SE	*T*	*p*
Acacia pied barbet/Ashy tit	−25.46	10.06	−2.531	0.094
Acacia pied barbet/Pygmy falcon	0.415	7.30	0.057	1.000
Acacia pied barbet/Scaly‐feathered weaver	−51.016	7.04	−7.248	< 0.001
Acacia pied barbet/Sociable weaver	19.415	4.85	−4.007	0.001
Ashy tit/Pygmy falcon	25.877	9.60	2.695	0.063
Ashy tit/Scaly‐feathered weaver	−25.554	10.24	−2.496	0.102
Ashy tit/Sociable weaver	6.047	8.24	0.734	0.948
Pygmy falcon/Scaly‐feathered weaver	−51.430	7.96	−6.460	< 0.001
Pygmy falcon/Sociable weaver	−19.829	5.80	−3.417	0.009
Scaly‐feathered weaver/Sociable weaver	31.601	5.85	5.401	< 0.001
